# Multi-omics Study of Planobispora rosea, Producer of the Thiopeptide Antibiotic GE2270A

**DOI:** 10.1128/mSystems.00341-21

**Published:** 2021-06-22

**Authors:** Francesco Del Carratore, Marianna Iorio, Mercedes Pérez-Bonilla, Kamila Schmidt, Rosario Pérez-Redondo, Margherita Sosio, Sandy J. Macdonald, Ivan S. Gyulev, Areti Tsigkinopoulou, Gavin H. Thomas, Olga Genilloud, Antonio Rodríguez-García, Stefano Donadio, Rainer Breitling, Eriko Takano

**Affiliations:** a Manchester Institute of Biotechnology, Faculty of Science and Engineering, University of Manchester, Manchester, United Kingdom; b NAICONS Srl, Milan, Italy; c Fundación MEDINA, Parque Tecnológico Ciencias de la Salud, Granada, Spain; d INBIOTEC Instituto de Biotecnología de León, León, Spain; e Department of Biology, University of York, Heslington, York, United Kingdom; f DTU Biosustain, Novo Nordisk Foundation Center for Biosustainability, Technical University of Denmark, Kongens Lyngby, Denmark; University of California, Berkeley

**Keywords:** thiopeptide, metabolomics, transcriptomics, genome-scale metabolic modelling, secondary metabolism, GE2270A

## Abstract

Planobispora rosea is the natural producer of the potent thiopeptide antibiotic GE2270A. Here, we present the results of a metabolomics and transcriptomics analysis of P. rosea during production of GE2270A. The data generated provides useful insights into the biology of this genetically intractable bacterium. We characterize the details of the shutdown of protein biosynthesis and the respiratory chain associated with the end of the exponential growth phase. We also provide the first description of the phosphate regulon in *P. rosea*. Based on the transcriptomics data, we show that both phosphate and iron are limiting *P. rosea* growth in our experimental conditions. Additionally, we identified and validated a new biosynthetic gene cluster associated with the production of the siderophores benarthin and dibenarthin in *P. rosea*. Together, the metabolomics and transcriptomics data are used to inform and refine the very first genome-scale metabolic model for *P. rosea*, which will be a valuable framework for the interpretation of future studies of the biology of this interesting but poorly characterized species.

**IMPORTANCE** Planobispora rosea is a genetically intractable bacterium used for the production of GE2270A on an industrial scale. GE2270A is a potent thiopeptide antibiotic currently used as a precursor for the synthesis of two compounds under clinical studies for the treatment of Clostridium difficile infection and acne. Here, we present the very first systematic multi-omics investigation of this important bacterium, which provides a much-needed detailed picture of the dynamics of metabolism of *P. rosea* while producing GE2270A.

**Author Video**: An author video summary of this article is available.

## INTRODUCTION

Planobispora rosea is a soil-dwelling, genetically intractable bacterium classified in the family *Streptosporangiaceae*. P. rosea is the first described producer of the antibiotic GE2270A (1), a molecule later detected in another member of the *Streptosporangiaceae*, *Nonomuraea* sp. strain WU8817 (2). This compound is a thiopeptide having potent activity against Gram-positive pathogens by targeting the elongation factor Tu (EF-Tu) (1). GE2270A is a member of the ribosomally synthesized and posttranslationally modified peptide (RiPP) class of natural products. The biosynthesis of RiPPs starts from the synthesis of a longer precursor peptide encoded by a structural gene (*pbtA* in the case of GE2270A). The precursor peptide consists of a leader peptide followed by the core peptide. During the biosynthesis, the precursor peptide undergoes a series of biochemical modifications before undergoing a specific cleavage, where the leader peptide is removed and the mature RiPP (i.e., modified core peptide) is exported (3). The experimental characterization of the *pbt* cluster has been attempted through the heterologous expression in Streptomyces coelicolor (4) and in *Nonomuraea* ATCC 39727 (5). GE2270A is considered to be a very promising natural product, mostly due to the fact that it has been successfully employed as a precursor for the semisynthesis of two compounds, LFF-571 and CB-06-01, currently under clinical studies. The most advanced LFF-571 has been investigated for the treatment of Clostridium difficile infections (6), although its development has been discontinued (7). CB-06-01 is currently considered a topical treatment for acne (8–10). P. rosea is currently used for the production of GE2270A on an industrial scale, and a deeper understanding of the metabolism of this strain in the context of GE2270A production is particularly important when trying to optimize the production of the target compounds. Multi-omics approaches are one of the most powerful tools currently available for the study of industrial microorganisms (11), and they have been very successful in the analysis of secondary metabolite production (12). Members of the order *Streptosporangiales*, which includes *Planobispora*, have provided valuable antibiotics that are either on the market or in clinical trials (6, 13), as well as a number of first-in-class discoveries (14). Yet, only rudimentary or inefficient gene transfer systems are available for a few of those strains (15). To our knowledge, no systematic multi-omics investigation has been performed for any member of this order, and it is currently not known to what extent models developed for well-studied *Actinobacteria* (e.g., few species of the genus *Streptomyces* [16]) can be used for distantly related actinobacteria. The genus *Planobispora* is poorly explored, with just four species formally described (17) and a single genome sequence available (18). Recent investigations have also suggested the need for a taxonomic reinvestigation of the *Planobispora* and *Planomonospora* genera (19). With these challenges in mind, here we use transcriptomics and metabolomics data to obtain a more detailed picture of the metabolism of this industrially important actinomycete while producing GE2270A.

## RESULTS

### Growth and antibiotic production during batch fermentation.

**FIG 1:**
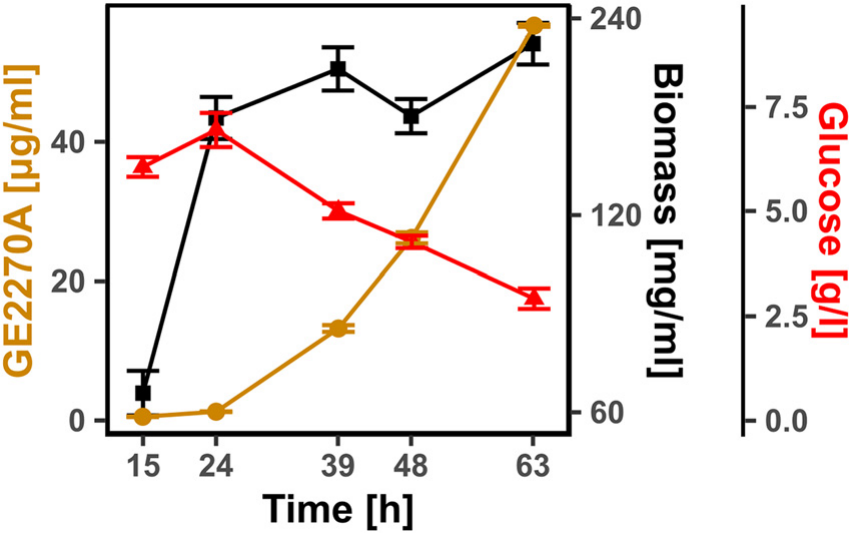
*P. rosea* fermentation data. Measured glucose, biomass, and GE2270A during fermentation of *P. rosea* in medium C. Biomass is shown on a logarithmic scale, while GE2270A and glucose concentrations are shown on a linear scale. The error bars represent the standard deviations calculated from three replicates.

Planobispora rosea ATCC 53733 was grown in medium C (see Materials and Methods). This medium provides good performance in terms of GE2270A production on a lab scale, and it is similar to the medium used for industrial production. Samples were collected in three replicates at 15, 24, 39, 48, and 63 h after inoculation for metabolomics and transcriptomics analysis. After 24 h, the culture entered stationary phase, following which GE2270A was found to accumulate to about 50 μg/ml at 63 h (Fig. 1). Throughout the fermentation, the amount of free glucose, both present in the starting medium and released from starch during microbial growth, remained high enough to sustain growth, suggesting that the culture was not carbon limited.

### Transcriptomics.

**FIG 2:**
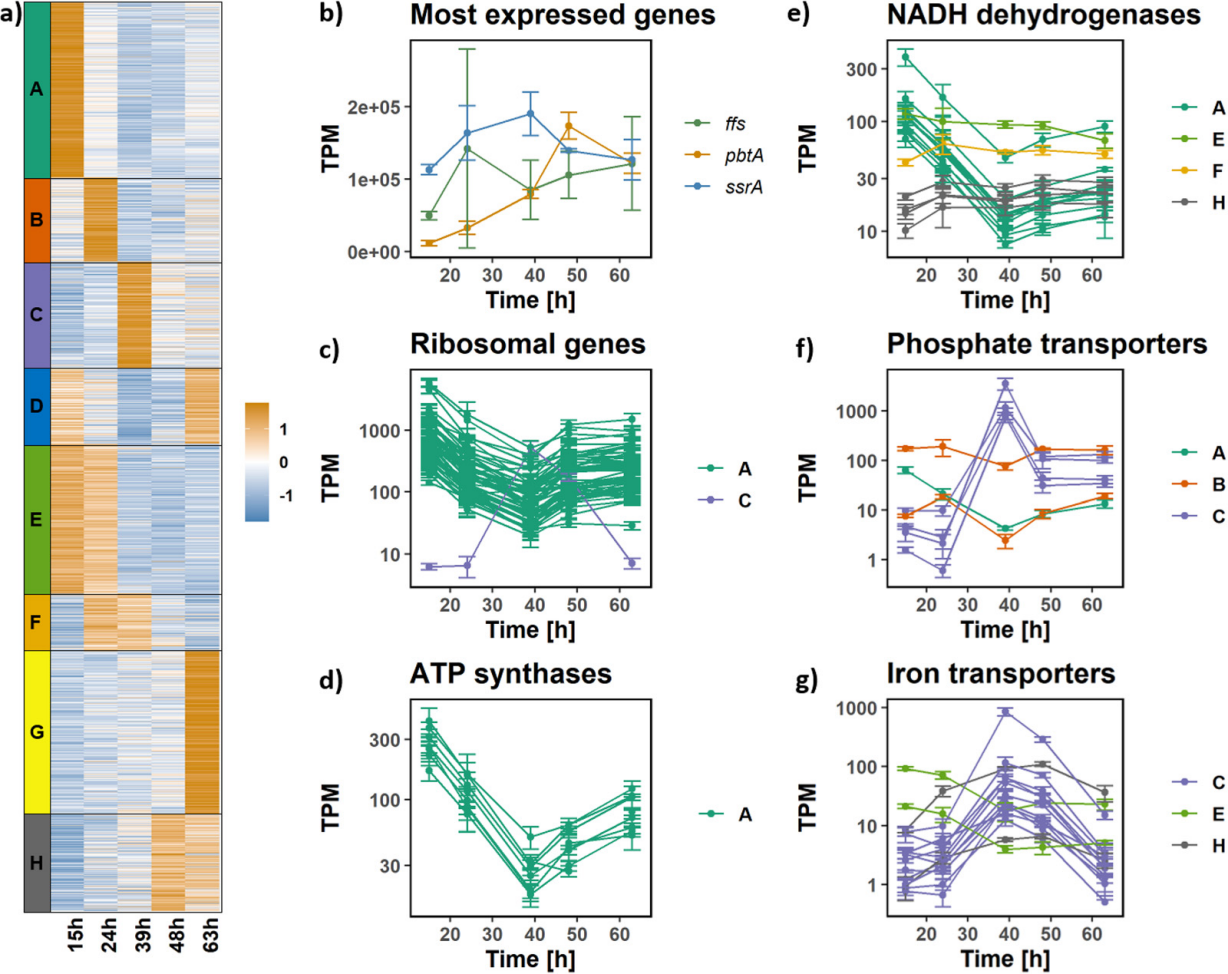
Transcriptome analysis: gene expression changes during fermentation. (a) Heatmap showing normalized TPM values associated with the genes identified as differentially expressed. The genes are clustered into eight groups with the k-means approach. (b) TPM values (on a linear scale) of the three most expressed genes during fermentation. (c) TPM values (on logarithmic scale) of the differentially expressed ribosomal genes. Most of them are part of the cluster A (green), with the exception of EV45_RS15600, which is part of cluster C (purple). (d) TPM values (on log scale) associated with all the differentially expressed genes annotated as ATP synthases. (e) TPM values (on log scale) associated with all the differentially expressed genes annotated as NADH dehydrogenase. (f) TPM values (on log scale) associated with all the differentially expressed genes involved in phosphate transport. (g) TPM values (on log scale) associated with all the differentially expressed genes involved in iron transport. The error bars represent the standard deviations calculated from three replicates.

The *P. rosea* genome was uploaded to the online tool MORF (https://morf-db.org/projects/TOPCAPI/P-rosea/genome/GCF_001696485.1/view) (20). This tool allows visualizing and exploring the transcriptomics data described in this section in the context of the genome sequence. The transcriptome profiles during the time course show that most of the *P. rosea* genome is transcribed during fermentation. Almost 73% of the 7,794 annotated genes found in the *P. rosea* genome showed an expression level higher than 5 transcripts per million (TPM), well above the limit of detection (see Fig. S1 and Supplementary file 1 at https://github.com/francescodc87/Multi-omics-study-of-Planobispora-rosea). All the genes with an average TPM value lower than 5 TPM at any time point are reported in Supplementary file 2 at https://github.com/francescodc87/Multi-omics-study-of-Planobispora-rosea. The largest region not transcribed spans from EV45_RS25620 to EV45_RS25970 and consists mostly of genes with unknown function. Apart from rRNA genes that were discarded from the analysis, the three most abundant transcripts are those of the *ssrA*, *ffs*, and *pbtA* genes (Fig. 2b). In a recent study, transcriptome sequencing (RNAseq) data were collected for the S. coelicolor M1146 strain cultivated using phosphate-limited fully defined medium (21). After excluding the rRNA genes from the analysis, the homologue of *ssrA* (SCOs02) was always the most expressed gene, and the homologue of *ffs* (SCOs03) was found in the top 30 most highly expressed genes in all experimental conditions considered. Both *ssrA* and *ffs* RNAs form extensive secondary structures, which are expected to provide increased cellular half-life and hence high levels in RNAseq data. The products of the *ssrA* and *ffs* genes are small RNAs that carry out housekeeping functions. Transfer messenger SsrA (locus tag EV45_RS05980), which is involved in releasing stalled ribosomes (22), displayed a constitutive expression with TPM values always higher than 1e5. The product of *ffs* (locus tag EV45_RS37010) is the RNA component of “the signal recognition particle” (the protein component corresponds to locus tag EV45_RS07710); similarly to what was observed for *ssrA*, its transcript level was always high and stayed roughly constant during fermentation. The signal recognition particle targets the nascent signal peptides of secreted or membrane proteins at translating ribosomes and deliver them to the plasma membrane (23). Remarkably, *pbtA*, the gene encoding the precursor peptide of GE2270A, is one of the three genes with the highest expression level. Its expression level peaked at 48 h when it was the most abundantly expressed gene (see further analyses below). Most of the loci annotated as tRNA showed a high expression level throughout fermentation. In fact when ranking the genes according to the maximum average gene expression level during fermentation, 53 out of the 63 tRNAs are found in the top 119 (probability of change [PC-value] = 7.83e−92 using the iterative Group Analysis [24]). However, the tRNA showing the highest gene expression level (EV45_RS35060, annotated as tRNA-Asp) is 3.4 times lower than that observed for *ssrA* at 63 h. Differentially expressed genes were identified with an analysis of variance (ANOVA) test, controlling the false discovery rate at 5%; 75.7% of the annotated genes found in the *P. rosea* genome showed a statistically significant change in expression during fermentation. To reveal trends in the gene expression, the selected genes were subjected to k-means clustering. The genes were clustered into eight groups (clusters A through H), each showing a specific trend, as shown in Fig. 2a. The composition of the clusters identified in this analysis is reported in Supplementary file 3 (https://github.com/francescodc87/Multi-omics-study-of-Planobispora-rosea). The clusters represent genes involved in the following major physiological switches occurring during transition to stationary phase and active antibiotic production.

### Shutdown of protein biosynthesis.

In the *P. rosea* genome, there are 64 genes encoding ribosomal proteins. The majority of them (56) showed a statistically significant change in expression level during fermentation. Interestingly, almost all of them followed a very similar trend (Fig. 2c): their expression peaked at 15 h and reached the lowest level at 39 h, which suggests a strong reduction in protein biosynthesis after 24 h. The only exception is a gene annotated as “30S ribosomal protein S4” (locus tag EV45_RS15600), which showed an opposite trend, peaking at 39 h. This protein shares only 45% identity with the “core” ribosomal protein S4; we suggest that it is not actually involved in forming the active ribosome but is a neofunctionalized ancient gene duplicate. BLAST searches detected close homologues only in members of the *Streptosporangiales*, suggesting an important and conserved role of this duplicate gene specifically within this order. It should be noted that the *P. rosea* genome does include a gene encoding a genuine ribosomal protein S4 (locus tag EV45_RS04055).

### Overexpression and subsequent downregulation of the respiratory chain.

Most genes involved in the respiratory chain followed the same pattern as the ribosomal proteins described in the previous section. With the sole exception of EV45_RS15870 (one of the two genes annotated as “F0F1 ATP synthase subunit gamma”), all the genes annotated as ATP synthases followed the trend of cluster A (Fig. 2d). Their expression peaked at 15 h and subsequently dropped reaching the minimum at 39 h. Then it slightly increased in the next two time points. A similar behavior is observed for the genes annotated as NADH dehydrogenases. The levels of expression of all the genes annotated as NADH dehydrogenases are shown in Fig. 2e. The data suggest that vigorous growth mostly stopped at 39 h, possibly due to depletion of some of the nutrients in the medium.

### Iron and phosphate transporters.

There are 658 genes involved in transport/secretion in the *P. rosea* genome, most of which (504) have been selected as differentially expressed during the fermentation. The complete transporter list can be found in Supplementary file 4 at https://github.com/francescodc87/Multi-omics-study-of-Planobispora-rosea. Interestingly, most of the genes involved in phosphate (Fig. 2f) and iron (Fig. 2g) transport showed very similar patterns, and they were grouped in cluster C. The same pattern is also observed for three of the four genes annotated as alkaline phosphatases (EV45_RS14425, EV45_RS14855, and EV45_RS22905) in the *P. rosea* genome and for one supposed molybdate transporter system (EV45_RS35765, EV45_RS35770, and EV45_RS35775), as shown in Fig. S2 (https://github.com/francescodc87/Multi-omics-study-of-Planobispora-rosea). In fact, their expression was relatively low in the first two time points and peaked at 39 h. This very similar expression pattern suggests a shared regulation system. For the alkaline phosphatases, this is not surprising, as they are part of the phosphate regulon (Table 1). It is thus possible that both phosphate and iron become the nutrients limiting growth at 39 h. This hypothesis has been tested with a supplementation experiment, and the results are discussed in the section “Supplementation of phosphate and iron.”

**TABLE 1:** List of members of the phosphate regulon identified in Planobispora rosea

Cluster^*a*^	*P. rosea* locus tag	Product	Symbol	S. coelicolor locus tag	Pho regulation^*b*^
C	EV45_RS03455	Phosphate transport system regulatory protein	*phoU*	SCO4228	Activation
C	EV45_RS03460	Two-component sensor histidine kinase	*phoR*	SCO4229	Activation
C	EV45_RS03465	DNA-binding response regulator	*phoP*	SCO4230	Activation
C	EV45_RS31875	Phosphate ABC transporter substrate-binding protein	*pstS*	SCO4142	Activation
C	EV45_RS31880	Phosphate ABC transporter permease PstC	*pstC*	SCO4141	Activation
C	EV45_RS31885	Phosphate ABC transporter permease PstA	*pstA*	SCO4140	Activation
C	EV45_RS31890	Phosphate ABC transporter ATP-binding protein	*pstB*	SCO4139	Activation
B	EV45_RS28300	Inorganic phosphate transporter	*pitH2*	SCO1845	Repression
A	EV45_RS03190	Inorganic phosphate transporter	*pitH1*	SCO4138	ND
A	EV45_RS03185	Pit accessory protein (DUF47)		SCO4137	ND
C	EV45_RS30455	Glycerophosphodiester phosphodiesterase	*glpQ1*	SCO1565	Activation
C	EV45_RS19780	Glycerophosphodiester phosphodiesterase	*glpQ2*	SCO1968	Activation
C	EV45_RS14425	Phospholipase D	*phoD*	SCO2068	Activation
C	EV45_RS22905	Alkaline phosphatase	*phoA*	SCO2286	Activation
C	EV45_RS14855	Alkaline phosphatase			Activation
C	EV45_RS34915	TAT-secreted putative phosphatase (DUF839)		SCO3790	Activation
C	EV45_RS33230	RNA degradosome polyphosphate kinase	*ppk*	SCO4145	Activation
C	EV45_RS33235	NUDIX hydrolase		SCO4143	Activation
C	EV45_RS15230	Hypothetical protein		SCO4877	Activation
C	EV45_RS15235	Glycosyltransferase family 2 protein		SCO4878	Activation
C	EV45_RS15225	Hypothetical protein		SCO4879	Activation
C	EV45_RS38880	Sialic acid synthase		SCO4880	Activation
C	EV45_RS15220	Hypothetical protein		SCO4882	Activation
B	EV45_RS12315	Type I glutamate-ammonia ligase	*glnA*	SCO2198	Repression
B	EV45_RS08445	Glutamine synthetase	*glnII*	SCO2210	Repression
B	EV45_RS07490	Ammonium transporter	*amtB*	SCO5583	Repression
B	EV45_RS07495	P-II family nitrogen regulator	*glnK*	SCO5584	Repression
B	EV45_RS07500	[Protein-PII] uridylyltransferase	*glnD*	SCO5585	Repression
C	EV45_RS04365	Glycerol-3-phosphate dehydrogenase/oxidase			Activation?

a The membership of each gene to the clusters identified by the k-means analysis and summarized in Fig. 2a.

b ND, not defined.

### Phosphate regulon.

The phosphate (Pho) regulon is a highly conserved regulatory system used by bacteria for the management of inorganic phosphate, first discovered in Escherichia coli (25). PhoP, part of the two-component system PhoR-PhoP, is the response regulator protein that activates or represses the genes of the regulon. The members of the Pho regulon in Streptomyces coelicolor are well-known (26–31). These S. coelicolor genes were used to identify the Pho regulon members in *P. rosea*, as reported in Table 1. Some orthologues were identified by sequence similarity (e.g., *phoP*); others were identified by synteny (e.g., *pstS*) or regulation pattern (e.g., *pitH2*). The genes that appeared to be activated by PhoP in *P. rosea* are all members of cluster C. Conversely, all the genes apparently repressed by PhoP are members of cluster B. Additional details about the members of the Pho regulon are found in Supplementary file 5 at https://github.com/francescodc87/Multi-omics-study-of-Planobispora-rosea. Similarly to what was observed in *Streptomyces* sp., the two-component system *phoR-phoP* and the modulatory regulator gene *phoU* are clustered in *P. rosea*. In S. coelicolor, the intergenic region between *phoU* and *phoR* contains the PhoP binding sites. When S. coelicolor is starved of phosphate, PhoR phosphorylates PhoP, and then PhoP binds to the target gene binding sites. Upon DNA binding, PhoP can function as an activator, including the activation of its gene via the *phoU-phoR* intergenic region and the activation of phosphate transport genes of the Pst system among the most upregulated genes in the regulon. As indicated by the respective TPM plots (see Supplementary file 5 at https://github.com/francescodc87/Multi-omics-study-of-Planobispora-rosea), it is very likely that the same occurs in *P. rosea*. Both S. coelicolor and *P. rosea* have two *pit* transporter genes (*pitH1* and *pitH2*). In both organisms, these genes seem to have a different regulation mechanism. In fact, *pitH2*, but not *pitH1*, appears to be repressed by PhoP. Other Pho regulon members activated in S. coelicolor and other bacteria are the phosphate scavengers and the polyphosphate kinase genes. As shown in Table 1, orthologues of these genes are found in *P. rosea*. The transcription profiles of the putative biosynthesis genes of teichuronic acids (EV45_RS15230 to EV45_RS15220 [Table 1]) resemble those of PhoP-activated genes. The replacement of cell wall teichoic acids by phosphate-free teichuronic acids serves as a source of phosphate in Bacillus subtilis (32). This mechanism is likely conserved in S. coelicolor (27) as well as in *P. rosea*. In S. coelicolor, the response to phosphate limitation is coordinated with the regulation of nitrogen assimilation. PhoP represses the transcription of nitrogen genes by its binding to the *glnR* promoter, encoding the major nitrogen regulator, to the promoters of *glnA* and *glnII*, encoding two glutamine synthetases, and to the promoter of the *amtB-glnK-glnD* operon, encoding an ammonium transporter and nitrogen sensing/regulatory proteins (29). A similar mechanism of PhoP-mediated repression appears to be conserved in *P. rosea*. Other members of cluster C may be activated by the Pho regulon, some of them involved in central metabolism (e.g., EV45_RS04365).

### Nitrogen metabolism.

The RNAseq data suggest that despite plentiful nitrogen sources being available in the complex medium, the free ammonium concentration is low enough to induce ammonium scavenging genes during the fermentation, reflected in orthologues of many genes under the positive control of GlnR in S. coelicolor being strongly induced (see Fig. S3a at https://github.com/francescodc87/Multi-omics-study-of-Planobispora-rosea) (33, 34). At 24 h, a strong increase in the expression levels of the *amtB* ammonium transporter gene (see Fig. S3b at https://github.com/francescodc87/Multi-omics-study-of-Planobispora-rosea) was detected, along with genes for high-affinity ammonium assimilation enzymes, namely, glutamine synthetase encoded by *glnA* and *glnII* (see Fig. S3c at https://github.com/francescodc87/Multi-omics-study-of-Planobispora-rosea), as well as genes encoding enzymes for release of ammonia from urea (see Fig. S3d at https://github.com/francescodc87/Multi-omics-study-of-Planobispora-rosea) and nitrite (see Fig. S3e at https://github.com/francescodc87/Multi-omics-study-of-Planobispora-rosea). As expected, the expression of the gene encoding glutamate dehydrogenase was reduced (see Fig. S3f at https://github.com/francescodc87/Multi-omics-study-of-Planobispora-rosea), consistent with low concentrations of free ammonia being insufficient to supply this enzyme. To protect against an ammonium surge, the *glnK* and *glnD* genes were also highly expressed (see Fig. S3b at https://github.com/francescodc87/Multi-omics-study-of-Planobispora-rosea), which likely are present to rapidly shut off AmtB activity (35). At later time points, the expression of these genes decreased, which is completely consistent with the induction of the PhoP phosphate limitation response (described above), which competes with GlnR for many of the same promoters (31). Interestingly, however, some of the genes which are not cross-regulated by PhoP remained increased in expression, including the nitrite reductase (see Fig. S3e at https://github.com/francescodc87/Multi-omics-study-of-Planobispora-rosea). The induction of the GlnR response, which our data suggest, behaves very similarly to that known in S. coelicolor. This is also consistent with its primary assimilated form, glutamine, showing an initial low extracellular concentration (see Fig. 6d). Consistent with the expression levels of glutamine synthetase genes decreasing after 24 h (see Fig. S3c at https://github.com/francescodc87/Multi-omics-study-of-Planobispora-rosea) as the culture transitions to phosphate limitation, the intracellular glutamate concentration increased significantly between 24 and 39 h and stayed roughly constant afterwards (see Fig. 6e).

### Expression of GE2270A biosynthetic gene cluster.

**FIG 3:**
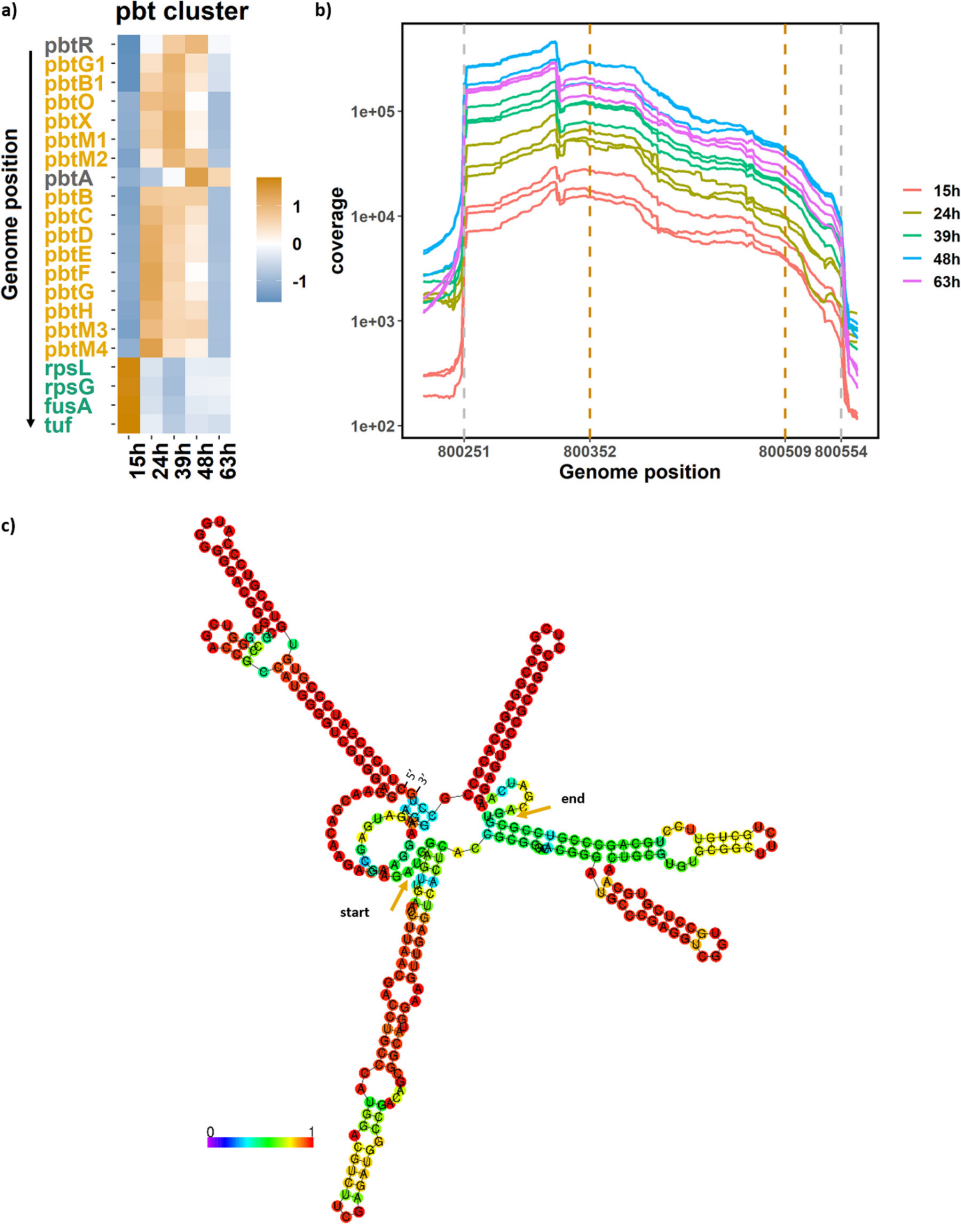
Gene expression of the *pbt* biosynthetic gene cluster. (a) Heatmap showing normalized TPM values associated with the GE2270A BGC. The gene names are color coded according to their pattern (green for cluster A, orange for cluster F, and gray for cluster H). (b) Coverage graph for *pbtA* obtained from the RNAseq. Gray dotted lines indicate predicted transcription start and end sites, and orange lines indicate the borders of the *pbtA* open reading frame. (c) Predicted secondary structure of the *pbtA* mRNA. Colors indicate base pair probabilities. Orange arrows indicate the translation start and end sites. 5′ and 3′ ends are indicated in the figure.

The RNAseq data collected in this study provide additional insights on the temporal expression levels observed for the genes involved in the biosynthesis of GE2270A. The *pbt* biosynthetic gene cluster (BGC) lies in the core region of the *P. rosea* genome. Interestingly, the genes found in the flanking region of this BGC include two ribosomal proteins (*rpsL* and *rpsG*) and two elongation factors (*fusA* and *tuf*). The expression of the *pbt* genes together with a selection of flanking genes is shown in Fig. 3a. The four genes in the flanking region included in the figure, followed the same pattern observed for most ribosomal proteins (cluster A), while most *pbt* genes followed the pattern observed in cluster F, and their expression peaked between 24 and 39 h. As already mentioned, *pbtA* is one of the three most highly expressed genes in the *P. rosea* genome (Fig. 2b), and it followed a different trend compared to the rest of the cluster. The expression pattern followed the general trend that would be expected for a just-in-time production process. The biosynthetic assembly line is put together first (early expression of the biosynthetic genes), and only when a substantial amount of all enzymes is available, the precursor peptide is made available at high levels. This avoids the accumulation of partly processed intermediates, which might be toxic or prone to be subjected to unwanted side reactions. Moreover, a just-in-time transcription strategy would reach the production goal while minimizing the total enzyme production (36–39).

From the RNAseq data, one can use the coverage information to identify the transcription start site for the *pbtA* gene (Fig. 3b). Transcription likely starts with the sequence CTTCG, 100 nucleotides upstream of the *pbtA* start codon and ends by a stem-loop situated soon after the *pbtA* stop codon. The translation start site automatically annotated for the *pbtA* gene is found in position 800343. However, identification of the leader peptide component of the PbtA peptide by mass spectrometry suggested that the correct translation start site is found in position 800352 (unpublished data). This is also supported by the presence of a strong Shine-Dalgarno sequence located 8 bp upstream to the start codon (AGGAGA). Using the RNAfold web server (40), it is possible to predict the optimal secondary structure of the *pbtA* mRNA with minimum free energy (Fig. 3c). Such structure includes four predicted hairpins, two of which occur right after the translation start and end sites. This secondary structure may explain the high TPMs observed for *pbtA*, which might be due to a combination of effective transcription and, especially, high RNA stability. We are currently investigating whether the observed high levels and possible secondary structures of *pbtA* mRNA have any impact on its translation into the precursor peptide.

### Expression of other predicted biosynthetic gene clusters.

The *P. rosea* genome was processed with the latest version of the antiSMASH platform (41) using default parameters. This led to the identification of 27 potential BGCs, one more than previously reported (18). A summary of the detected BGCs is reported in Table S1 at https://github.com/francescodc87/Multi-omics-study-of-Planobispora-rosea. The complete output of the antiSMASH analysis of the *P. rosea* genome can be found in Supplementary file 6 at https://github.com/francescodc87/Multi-omics-study-of-Planobispora-rosea. The gene expression associated with these clusters has been captured by the RNAseq data obtained in this study. Interestingly, there are three BGCs (those associated with regions 20, 21, and 27 in Table S1 [https://github.com/francescodc87/Multi-omics-study-of-Planobispora-rosea]), in addition to the *pbt* cluster, that seem to be expressed at substantial levels. The expression levels of regions 20 and 27 peaked at 39 h, while the expression levels for region 21 were constantly high during fermentation. The gene expression profiles of two of these clusters are shown in Fig. S4 (https://github.com/francescodc87/Multi-omics-study-of-Planobispora-rosea). The expression levels of the genes associated with the cluster found in the region 21 were relatively high and constant after the 24-h time point. Conversely, the expression of the genes found in region 20 and region 27 (not shown) peaked at 39 h, when protein biosynthesis largely stopped, a downregulation of the respiratory chain is observed, and the phosphate regulon seemed active. As mentioned before, at the same time an upregulation of iron transporters is also observed. It is possible that the upregulation of these biosynthetic clusters is observed as a response to phosphate or iron depletion. This hypothesis becomes very intriguing when considering that the BGCs in region 20 and in region 27 show a significant similarity with two known BGCs responsible for the biosynthesis of two siderophores (streptobactin and erythrochelin, respectively). An analysis of region 27 and the corresponding metabolite will be reported elsewhere. The product of region 20 will be discussed below.

### Supplementation of phosphate and iron.

**FIG 4:**
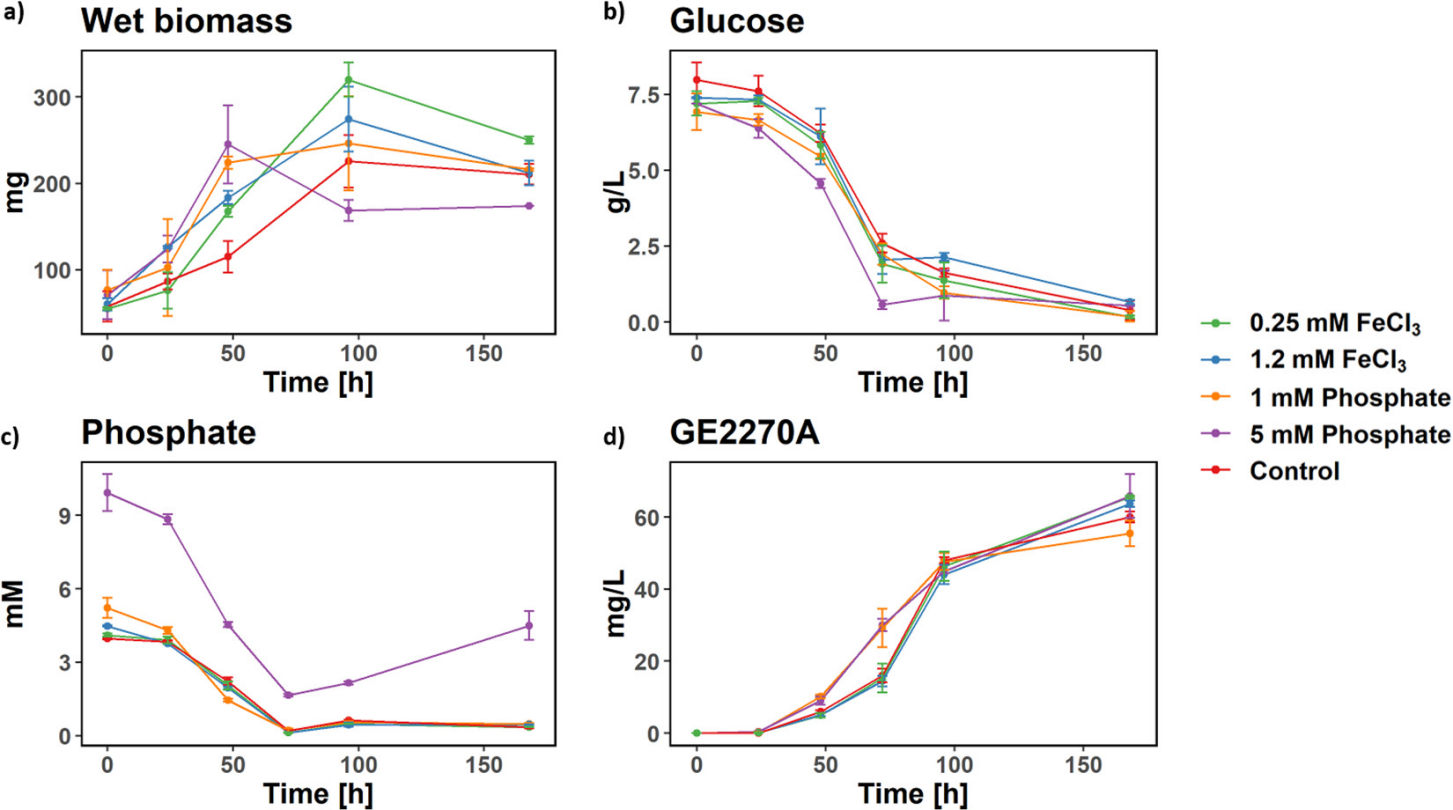
Effect of phosphate and iron supplementation. For all five experimental conditions, wet biomass (a), glucose (b), phosphate (c), and GE2270A (d) levels were measured every 24 h. The error bars represent the standard deviations calculated from two replicates.

As mentioned in the previous section, the transcriptomics data suggest that, under these specific growth conditions, phosphate and iron could be growth-limiting factors. In order to test this hypothesis, *P. rosea* was cultivated at 100-ml scale in duplicates containing medium C supplemented with either 1 or 5 mM phosphate or with 0.25 or 1.2 mM FeCl_3_, monitoring GE2270A, glucose, phosphate, and wet biomass levels every 24 h up to 7 days. Supplementation of either phosphate or iron had a clear effect on biomass production (Fig. 4a): at 48 h, the biomass observed in each experimental condition was significantly higher than in the control experiment, with no significant difference between the two phosphate or iron concentrations. This observation confirms the hypothesis generated from the transcriptomics data. Interestingly, phosphate supplementation at either 5 or 1 mM led to a slightly earlier GE2270A production and higher levels at 48 and 72 h in comparison with the control or the iron-supplemented conditions (Fig. 4d). However, this effect disappeared at 96 h. Glucose consumption appears to be very similar across experimental conditions, with the possible exception of 5 mM phosphate supplementation. In this condition, glucose uptake is significantly higher until 72 h and stops afterwards. Interestingly, this increased glucose uptake is associated with a significantly lower final biomass of the culture. Recently, the regulation of some genes encoding glycolytic enzymes by PhoP has been suggested for S. coelicolor and Streptomyces lividans, some genes being positively regulated, others negatively (42). An analogous influence of PhoP regulation on aspects of glycolysis might also be present in *P. rosea*, and the details of this interaction will be an interesting subject for future study.

### Metabolomics.

With the aim of having a clearer understanding of the physiological rearrangements resulting from the transcriptome dynamics of *P. rosea* during fermentation, different metabolomics analyses were performed. Using high-resolution mass spectrometry quadrupole time of flight (HRMS-QTOF) mass spectrometry, a targeted metabolomics analysis on the whole-broth acetonitrile extracts was performed. Moreover, untargeted metabolomics experiments using a QExactive plus were performed for the analysis of the exometabolome (metabolites found in the extracellular environment) and the endometabolome (metabolites found inside the cells). All samples for all metabolomics experiments were collected at the same time as the samples for transcriptomics analysis (15, 24, 39, 48, and 63 h after inoculation). More details are given in Materials and Methods.

### Whole-broth analysis.

**FIG 5:**
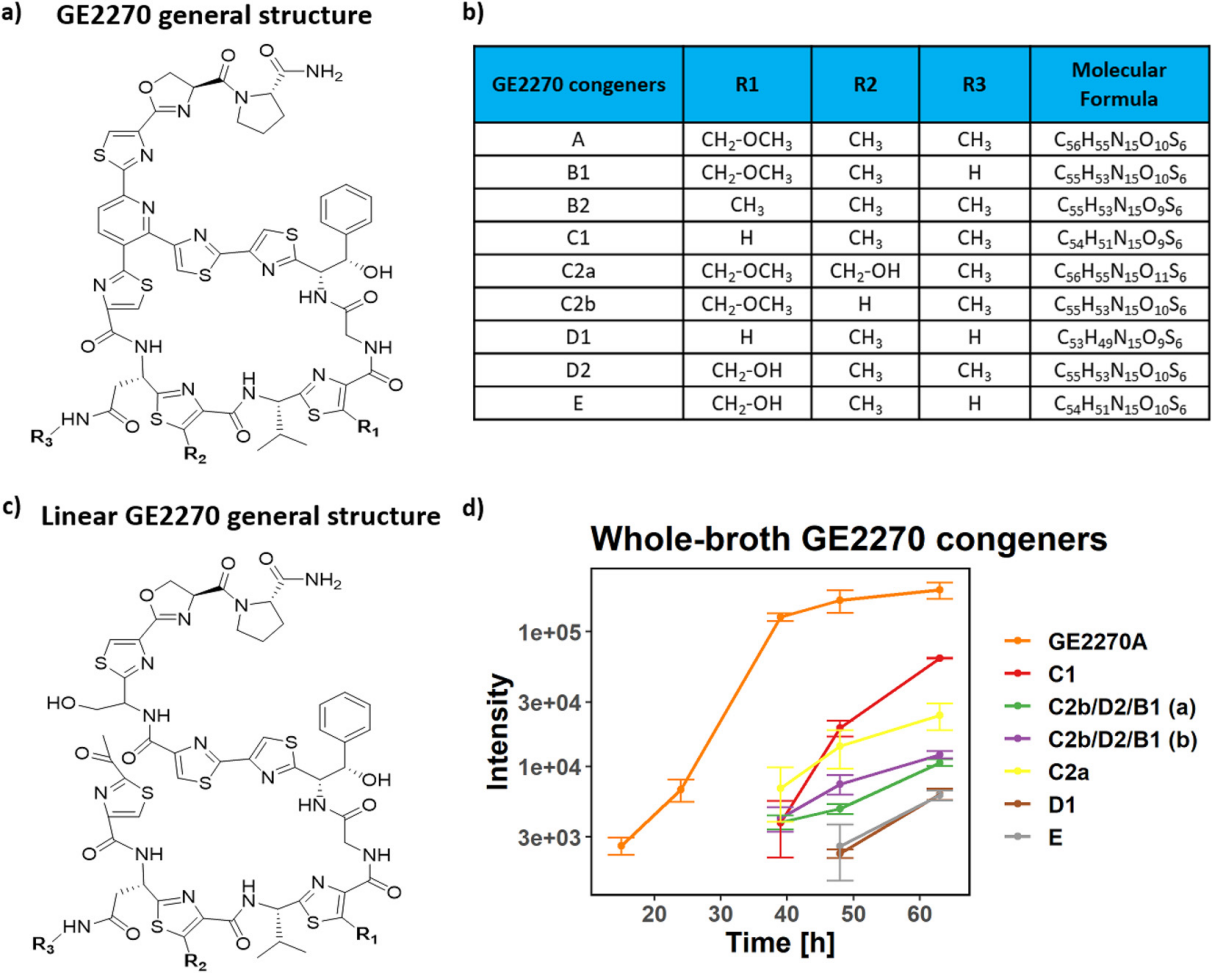
Targeted metabolomics analysis of whole-broth acetonitrile extracts. (a) General structure of the GE2270A congeners. (b) List of GE2270A congeners detected in the extracts of Planobispora rosea. (c) General structure of the linear/hydrated congeners. (d) Intensities over time associated with peaks of the GE2270A congeners in the acetonitrile extract of the whole broth. Intensities are shown on a logarithmic scale. The error bars represent the standard deviations calculated from three replicates.

The quantification of GE2270A in the whole-broth acetonitrile extracts was performed by liquid chromatography, UV detection, and mass spectrometry (LC-UV-MS) analysis. The quantification at 310 nm revealed that the extraction of this thiazolyl peptide was efficient when using acetonitrile as the extraction solvent. The concentration of GE2270A in the acetonitrile extract was 54 mg/liter at 48 h. This justified the use of the acetonitrile extraction for the analysis of the whole-broth samples. The acetonitrile extracts were analyzed using HRMS-QTOF instrumentation. In a targeted experiment, all the congeners reported to be produced together with GE2270A by *P. rosea* (1) were monitored and summarized in Fig. 5b. The intensity over time for all the congeners detected in the samples is shown in Fig. 5d. As expected, GE2270A is the main one, followed by the peak annotated as congener C1. The peaks associated with congeners C2a, D1, and C2b, D2 and/or B1 were detected at significantly lower levels, whereas congener B2 was detected only at trace levels. As shown in Fig. 5b, congeners C2b, D2, and B1 have the same chemical formula, and it is not possible to discriminate between them with mass spectrometry. The untargeted metabolomics experiment performed on the acetonitrile extracts detected several mass-to-charge ratios associated with a total of 67 metabolites for most of which no putative annotation is available. These results are reported in Supplementary material and Fig. S5 at https://github.com/francescodc87/Multi-omics-study-of-Planobispora-rosea.

### Exometabolome analysis.

**FIG 6:**
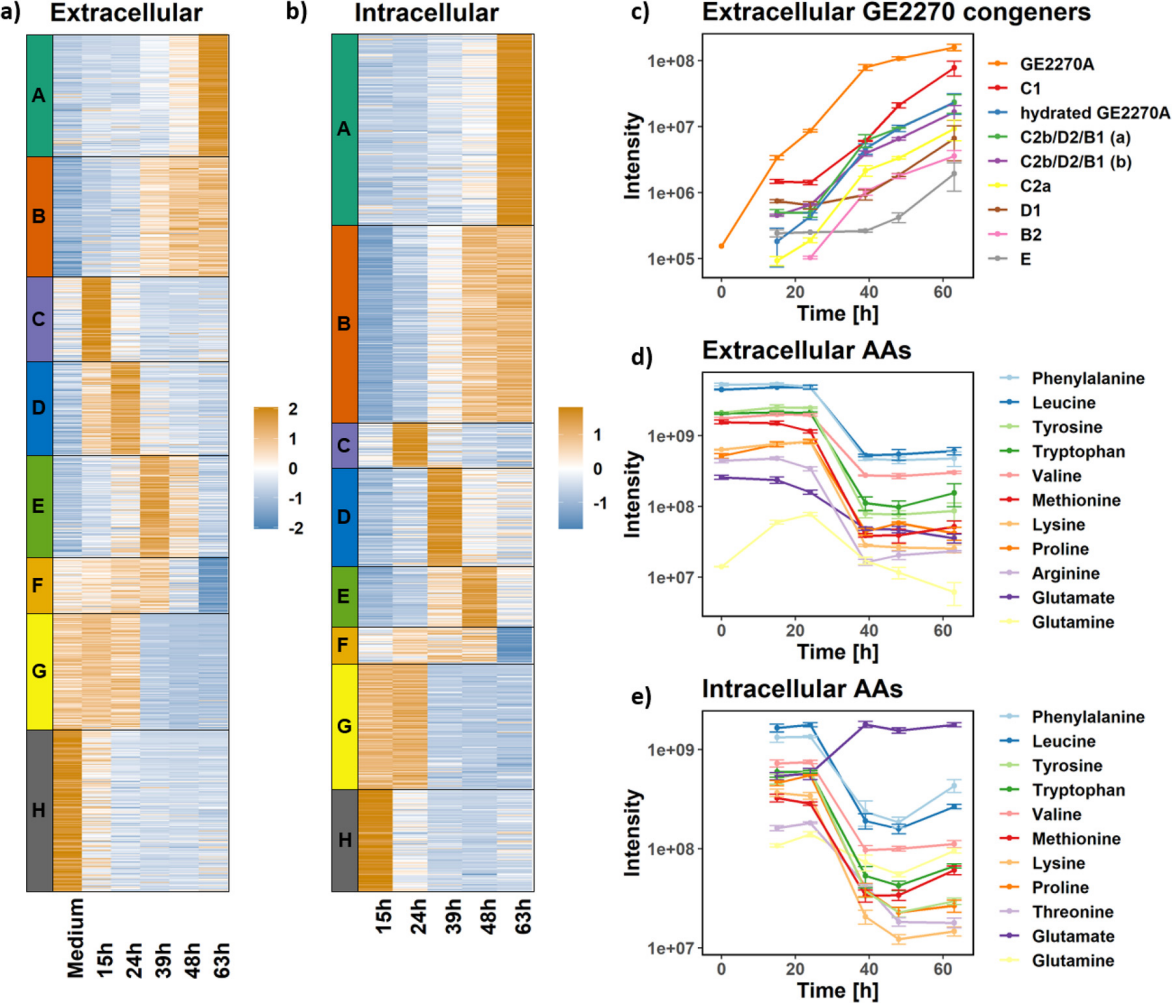
Untargeted metabolomics data for exo- and endometabolome. (a) Heatmap showing normalized intensity values associated with the peaks, detected in the extracellular environment in the positive mode, whose levels show a statistically significant change during fermentation. The peaks are clustered into eight groups with the k-means approach. (b) Heatmap showing normalized intensity values associated with the peaks, detected in the intracellular environment in the positive mode, whose levels show a statistically significant change during fermentation. The peaks are clustered into eight groups with the k-means approach. (c) Intensities over time associated with the peaks for which the most likely annotation is one of the GE2270A congeners. The hydrated GE2270A is not a real metabolite, but an artifact of the LC-MS analysis. Data are shown on a logarithmic scale. (d) Intensities over time associated with the peaks for which the most likely annotation is an amino acid (AA) in the extracellular environment. The error bars represent the standards deviation calculated from three replicates. Data are shown on a logarithmic scale. (e) Intensities over time associated with the peaks for which the most likely annotation is an amino acid in the intracellular environment. Data are shown on a logarithmic scale. The error bars represent the standard deviations calculated from three replicates.

When analyzing the untargeted metabolomics data obtained for the extracellular fraction, we detected 2,174 main peaks in positive mode and 1,213 in negative mode. A GE2270A standard sample was analyzed together with the biological samples. As shown in Fig. S6a at https://github.com/francescodc87/Multi-omics-study-of-Planobispora-rosea, the standard allowed the certain identification of the peak associated with GE2270A. In order to identify the metabolites showing a statistically significant change in abundance during fermentation, an ANOVA test on the normalized values was performed. The obtained *P* values were corrected for multiple testing using the Benjamini-Hochberg method (43) to control the false discovery rate at 5%. Using the additional requirement of a maximum average log_2_ fold change greater than 1, we could see 64% of the detected peaks in positive mode showing a statistically significant change during fermentation (68% in negative mode). To reveal trends in the abundance of the metabolites detected in positive mode, the peaks were clustered into eight groups using the k-means clustering approach (Fig. 6a and Supplementary file 7 at https://github.com/francescodc87/Multi-omics-study-of-Planobispora-rosea). Each group shows a specific trend: cluster A and cluster B contain all the metabolites that accumulated in the extracellular environment throughout fermentation. As shown in Fig. 6c, some of these compounds were produced by *P. rosea*, such as the GE2270A congeners described in Fig. 5a to c, which were also detected in the whole-broth acetonitrile extracts (see Supplementary material at https://github.com/francescodc87/Multi-omics-study-of-Planobispora-rosea). Clusters C to F all include metabolites which peaked between 15 and 48 h. In particular, cluster E contains two features (identifier [ID] 582 with *m/z* 412.1822 and ID 272 with *m/z* 403.1774) corresponding to benarthin and dibenarthin. While the dibenarthin feature is only putatively annotated, the identification of the benarthin feature has been confirmed through tandem mass spectrometry (MS2) fragmentation pattern and UV spectrum, as shown in Fig. S7 (https://github.com/francescodc87/Multi-omics-study-of-Planobispora-rosea). The possible association of these metabolites with the BGC identified in region 20 is discussed below. The last two clusters (G and H) include the metabolites decreasing in abundance during fermentation. These metabolites are most likely to be nutrients consumed by *P. rosea* during growth. All the metabolites putatively annotated as amino acids with high probability are shown in Fig. 6d. Almost all of them are members of cluster G, with the exception of glutamine (cluster D). Their concentration was more or less constant until 24 h and rapidly dropped between 24 and 39 h. According to the transcriptomics data, this happened at the same time when biomass accumulation and protein biosynthesis largely stopped, and a downregulation of the respiratory chain was observed. The same approach described here was also applied for the statistical analysis of the untargeted metabolomics data acquired in negative mode. As shown in Fig. S8 (https://github.com/francescodc87/Multi-omics-study-of-Planobispora-rosea), the clusters identified by the k-means approach in the data acquired in negative mode are very similar to the ones found in positive mode. Expectedly, a significant lower number of metabolites were measured in negative mode, and the results are reported in Supplementary file 8 (https://github.com/francescodc87/Multi-omics-study-of-Planobispora-rosea).

### Endometabolome analysis.

While the exometabolome profiles identified changes in the uptake and secretion fluxes of key metabolites, observing the changes in concentration in the intracellular fraction provides a better understanding of the metabolic changes underlying these dynamics. From the untargeted metabolomics data, we obtained 1,813 main peaks in positive mode and 593 in negative mode. Figure S6b at https://github.com/francescodc87/Multi-omics-study-of-Planobispora-rosea shows that the main congener GE2270A was detected in the intracellular environment at increasing levels throughout fermentation. The metabolites showing a statistically significant change in abundance during fermentation were identified using the same approach used for the exometabolome. To reveal trends in the levels of the metabolites selected in positive mode, the peaks were again clustered into eight groups using the k-means clustering approach (see Supplementary file 9 and Supplementary file 10 at https://github.com/francescodc87/Multi-omics-study-of-Planobispora-rosea). The trends found in the metabolomics data acquired in positive mode are shown in Fig. 6b. Cluster A and cluster B contain all the metabolites that accumulated inside the cells during fermentation. These two groups include all the detected congeners produced by the *pbt* cluster (see Fig. S6c at https://github.com/francescodc87/Multi-omics-study-of-Planobispora-rosea). Also in the case of the endometabolome, benarthin and dibenarthin are detected (ID 1084 and 818, respectively), and they follow the trend associated with cluster D. Similarly to what was observed in the exometabolome, most of the metabolites putatively annotated as amino acids with high probability are members of cluster G, and their concentration decreases between 24 and 39 h (Fig. 6e). Glutamate is the only exception. In fact, it follows the opposite trends of the other amino acids, and its levels sharply increase between 24 and 39 h. The corresponding, very similar results for data acquired in negative mode are shown in Fig. S9 (https://github.com/francescodc87/Multi-omics-study-of-Planobispora-rosea). Also in this case, the main findings observed in positive mode are confirmed by the data acquired in the negative mode. Almost all the detected amino acids follow the same trends in both the exometabolome and endometabolome. The only exceptions are glutamine (in the exometabolome) and glutamate (in the endometabolome). This behavior could be related with the repression of the rearrangement of the nitrogen metabolism described above.

### Analysis of the BGC found in region 20.

**FIG 7:**
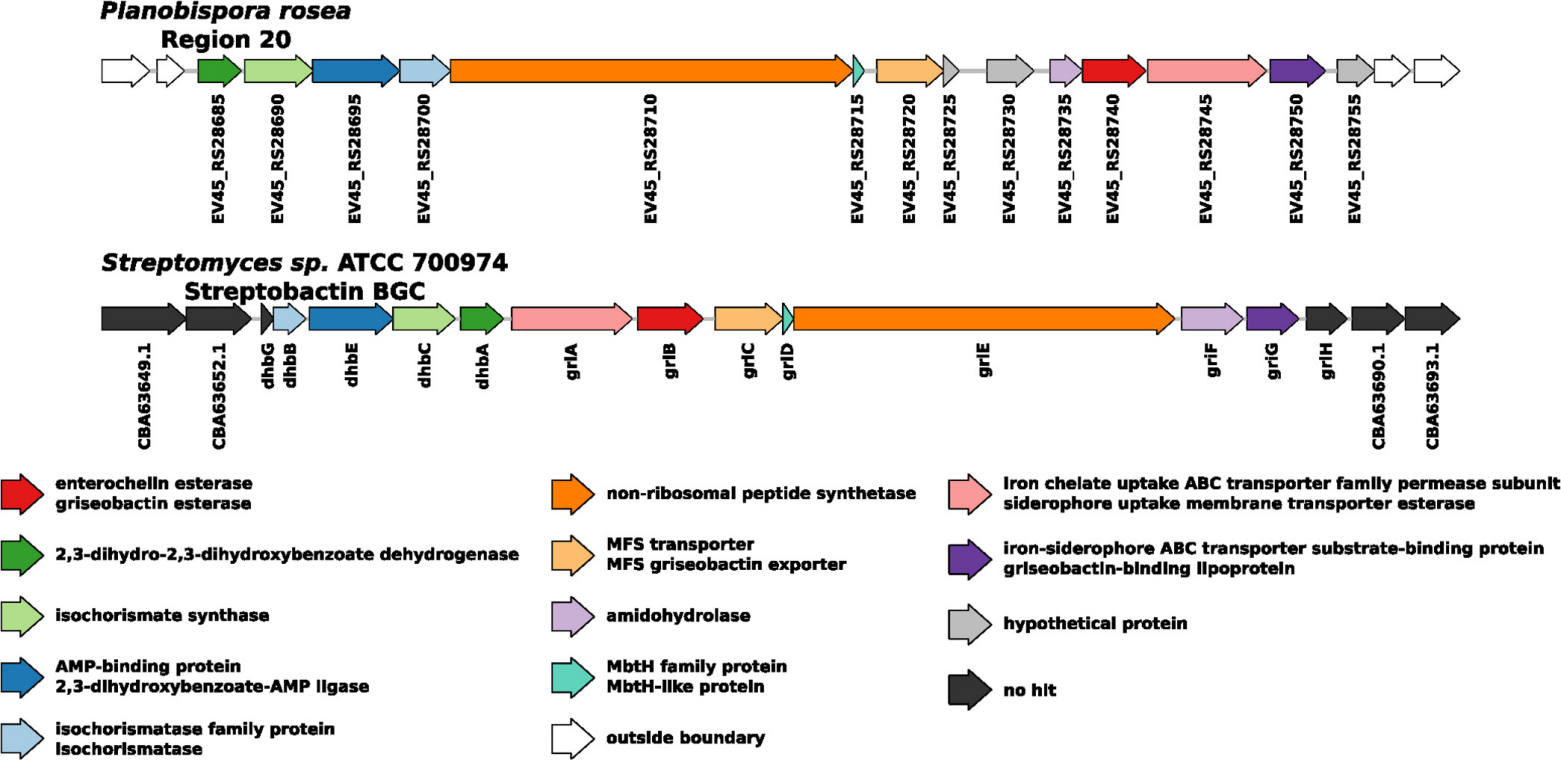
Region 20 versus streptobactin BGC. Comparison of the BGC found in *P. rosea* believed to be responsible for the production of benarthin and dibenarthin and the streptobactin biosynthetic gene cluster found in *Streptomyces* sp. ATCC 700974. Orthologue genes are represented by the same color. If orthologues show a different annotation in the two genomes, both annotations are reported in the legend.

As mentioned in the previous section, we were able to identify the presence of two metabolites: benarthin and dibenarthin. The dibenarthin feature has been putatively annotated with a high degree of certainty using the Integrated Probabilistic Annotation (IPA) approach (44). On the other hand, we were able to confirm the identification of benarthin through MS2 fragmentation pattern and UV spectrum as shown in Fig. S7 at https://github.com/francescodc87/Multi-omics-study-of-Planobispora-rosea. The concentration of these compounds peaked at 39 h and correlates with the gene expression observed for the BGC identified in region 20 (see Fig. S4a at https://github.com/francescodc87/Multi-omics-study-of-Planobispora-rosea). Both metabolites have been reported to be strictly associated with streptobactin (45), which is the trimeric form of benarthin, and according to the antiSMASH analysis (see Table S1 at https://github.com/francescodc87/Multi-omics-study-of-Planobispora-rosea), the biosynthetic cluster in region 20 shows a significant similarity with the streptobactin BGC found in *Streptomyces* sp. strain ATCC 700974. In Fig. 7, we show a direct comparison between the streptobactin BGC found in *Streptomyces* sp. ATCC 700974 and the BGC found in region 20. The borders of the latter BGC were identified using the transcriptomics data. All the data summarized here suggest that the cluster identified in region 20 is associated with the production of benarthin and dibenarthin in *P. rosea*.

### Genome-scale metabolic model.

A genome-scale metabolic model is not currently available for *P. rosea*. To facilitate the integration of transcriptome and metabolome data, we constructed a genome-scale metabolic model for *P. rosea* using comparative modeling, based on the related actinomycete S. coelicolor, for which we had previously developed a manually curated model (iAA1259 [46]), and refined this draft using the data collected here. Additional details on the building of this model can be found in Materials and Methods, and the model can be found as Supplementary file 11 at https://github.com/francescodc87/Multi-omics-study-of-Planobispora-rosea and on the MORFlux FBA Tool (https://morf-db.org/projects/TOPCAPI/P-rosea/metabolome/fba [20]). As previously mentioned, the replacement of cell wall teichoic acids with teichuronic acids during phosphate starvation is likely to be occurring in *P. rosea*. In order to represent this process in the genome-scale metabolic model, an alternative biomass reaction has been included in the model. The biosynthesis of teichuronic acid is missing in iAA1259, and it was manually added in the *P. rosea* model. The teichuronic acid considered is as a polymer containing *N*-acetylgalactosamine and d-glucuronic acid in equal proportions, initially isolated from Bacillus licheniformis 6346 (47). The metabolite defined in the model consists of 25 repeating units of the monomer. In order to explain its production, the model required the addition of the reaction for the biosynthesis of teichuronic acid from UDP-*N*-acetylglucosamine and UDP-d-glucuronate. The production of UDP-*N*-acetylglucosamine was obtained by the addition of the UDP-*N*-acetylglucosamine 4-epimerase reaction. In B. subtilis, this reaction is catalyzed by *galE* (48). An orthologue of this gene is present in the *P. rosea* genome (locus tag EV45_RS04840). In order to describe the phosphate starvation with the resulting genome-scale model, we devised an *in silico* experiment where we simulated the dynamic growth of *P. rosea* using dynamic flux balance analysis (dFBA). This method assumes that bacterial metabolism reaches an internal steady state almost immediately after a change in the extracellular environment. This allows the update of the extracellular concentrations based on the exchanges predicted by the model, which in turn update the uptake rates of the substrates needed for growth (49, 50). In this experiment, we considered a minimal medium where glucose is the only source of carbon (initial concentration, 222.2 mmol/liter), the initial concentration of inorganic phosphate is relatively low (4.6 mmol/liter), and the initial biomass concentration is 0.5 gDW/liter where gDW stands for grams in dry weight. No limit on the uptake of all the other necessary nutrients (e.g., minerals and ammonium) was considered. Based on the concentration of the extracellular inorganic phosphate ([pi]), two different conditions were considered. When [pi] was >0.1 mmol/liter, normal growth is considered: the maximum glucose and phosphate uptake is constrained based on the external concentration following Michaelis-Menten equations:

(1) $$v_{\text{glucose}}=\frac{V_{\text{max}}[\text{glucose}]}{K_{m}\;+\;[\text{glucose}]}$$

(2) $$v_{\text{pi}}=\frac{V_{\text{max}}[\text{pi}]}{K_{m}\;+\;[\text{pi}]}$$

where *v*_glucose_ and *v*_pi_ are the maximum uptake rates allowed at any time. During normal growth, teichoic acids are produced as per biomass composition, and the availability of this phosphate reserve is considered in this simulation. The phosphate threshold has been selected to be 0.1 mmol/liter, since this is the threshold experimentally observed for the activation of the Pho regulon in S. coelicolor (51). When [pi] goes below the threshold, the cells enter phosphate starvation: in the model, the alternative biomass reaction is considered, the biosynthesis of teichoic acids is blocked, the biosynthesis of teichuronic acids is allowed, the uptake of inorganic phosphate is blocked. Additionally, the maximum conversion rate for teichoic acids is constrained following a Michaelis-Menten equation:

(3) $$v_{\mathrm{teichoic}\text{acid}}=\frac{V_{\text{max}}[\mathrm{teichoic}\text{acid}]}{K_{m}\;+\;[\mathrm{teichoic}\text{acid}]}$$

**FIG 8:**
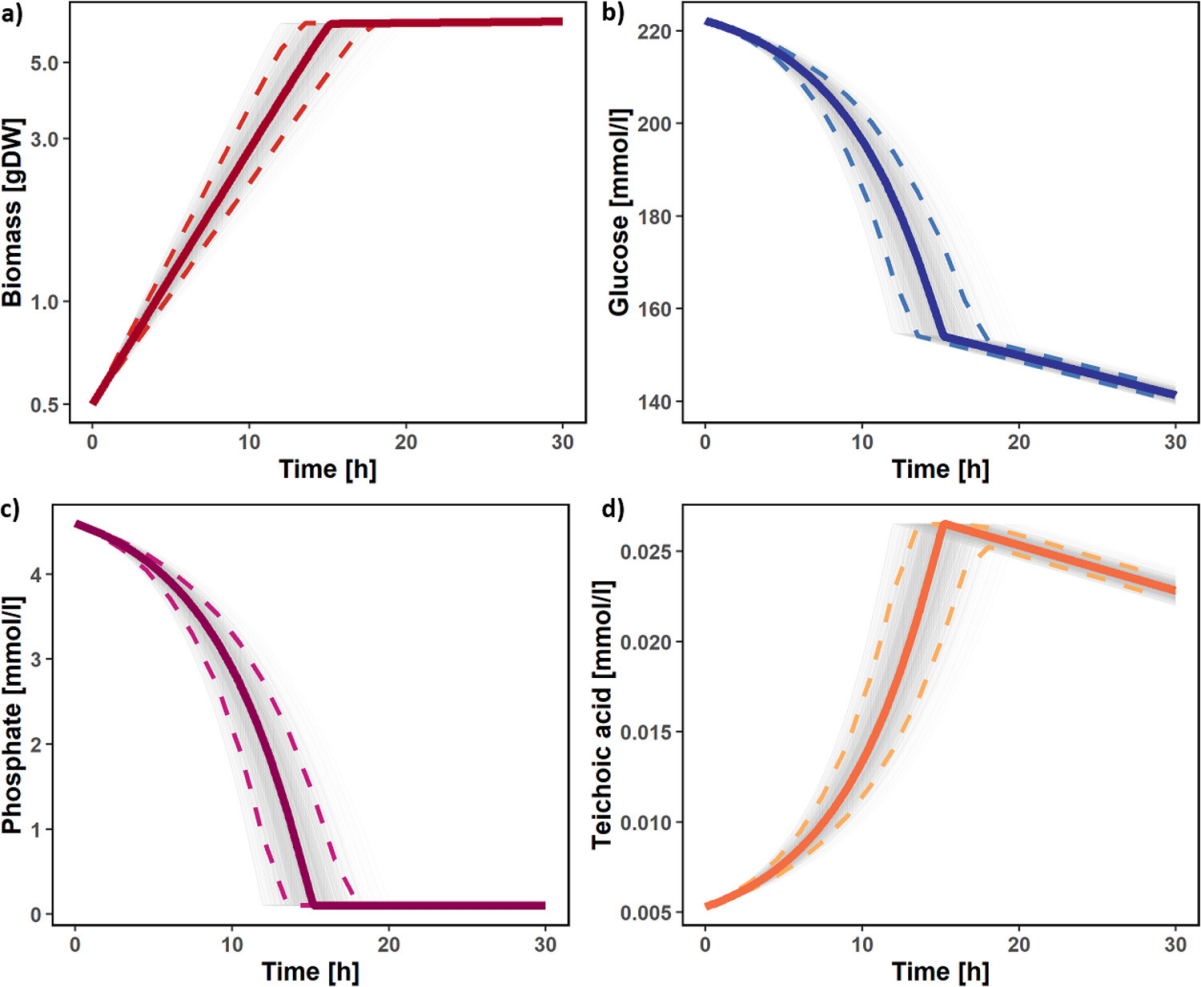
dFBA simulation during phosphate starvation. The graphs show the changes over time predicted with the dFBA approach for biomass (a), glucose (b), phosphate (c), and teichoic acids (d). Gray lines represent the results obtained for each of the 1,000 ensemble modeling simulations. Thick lines represent the medoid simulation, and the dashed lines represent the 95% confidence interval.

The maximum uptake of oxygen (*v*_O_2__) is also constrained throughout growth. Very little is known about the kinetics of the substitution of teichoic acids, during their degradation use as a phosphate reserve. We model this whole process with a simple reaction. In order to free up the phosphate present in one molecule of wall teichoic acid, this reaction forces the cell to substitute a molecule of teichoic acid with one molecule of teichuronic acid. The kinetic parameters used in equation 1, equation 2, and equation 3 and the maximum O_2_ uptake rate in *P. rosea* are not known. Nevertheless, we were able to define informative prior distributions for each of the parameters considered based on experimental values measured for related organisms following the protocol described by Tsigkinopoulou et al. (52). The definition of these distributions is described in the Materials and Methods. This allowed us to implement an ensemble modeling approach where a set of parameters was sampled from the distribution 1,000 times. Each parameter set was used to simulate the growth of *P. rosea* for 30 h in the medium previously defined with the dFBA, resulting in a rigorous assessment of the confidence interval of the flux predictions, despite the uncertainty about the exact kinetic parameter values. The results are shown in Fig. 8.

During the initial phase of growth, biomass accumulation appears to be exponential (Fig. 8a), and both phosphate and glucose are rapidly consumed (Fig. 8b and c). At the same time, the teichoic acids are accumulated as part of the biomass. This initial phase stops after 15 h (95% confidence interval, 13.3 to 17.8). This happens when the concentration of phosphate goes below the defined threshold. At this point, the teichoic acids begin to be consumed as a phosphate source (Fig. 8d), growth dramatically slows down, and consequently, the consumption of glucose slows down too. Given the highly similar overall enzyme content of a number of *Planobispora* species, including P. longispora, P. siamensis, and P. takensis (see Supplementary file 12 at https://github.com/francescodc87/Multi-omics-study-of-Planobispora-rosea), we can conclude that our *P. rosea* model will also serve as a strong starting point for similar models for other species within this genus.

## DISCUSSION

In this study, we present a comprehensive multi-omics study of the fermentation of Planobispora rosea while producing the thiopeptide antibiotic GE2270A. The data collected in this study greatly improved our understanding of the biology of this genetically intractable bacterium while providing insights into the expression of GE2270A biosynthesis genes. The transcriptomics data allowed the characterization of the shutdown of protein biosynthesis and the respiratory chain associated with the end of the exponential growth phase. For the first time, we reported a detailed description of the phosphate regulon in *P. rosea*. Analysis of the transcriptomics data showed that both iron and phosphate are growth limiting in the experimental condition used. This observation has been confirmed experimentally. Additionally, a new biosynthetic gene cluster has been identified and associated with the production of the siderophores, benarthin and dibenarthin. The predicted secondary structure of the *pbtA* mRNA suggests a possible strategy for achieving high levels of precursor peptides during RiPP biosynthesis. Once the processing enzymes are available, a stable and translatable mRNA is expected to ensure sufficient precursor peptide for further processing. To our knowledge, no equivalent studies have been performed in other RiPP producers, although our unpublished observations indicate that many precursor peptide mRNAs appear to have the potential to form secondary structures likely to confer stability. The data collected from the metabolomics experiments provided a clearer picture of the changes in the metabolism of *P. rosea* during fermentation and GE2270A production. Through the metabolomics experiments it was possible to observe the production dynamic of GE2270A with its congeners and the concentration dynamic of the main amino acids both in the extracellular and intracellular environment. The production of two siderophores (benarthin and dibenarthin) was detected by untargeted metabolomics from *P. rosea* for the first time. The BGC associated with their production was also identified in this study. Finally, both the transcriptomics and metabolomics data collected here were used in the refinement of the very first genome-scale metabolic model built for *P. rosea*. The model was also able to simulate the replacement of cell wall teichoic acids with teichuronic acids during phosphate starvation and its exploitation as phosphate storage by the bacterium. In conclusion, we show that even for a genetically intractable strain, multi-omics data can provide important insights into the biology of a microorganism of interest, using the most related model strains to highlight similarities and differences. In fact, the omics data as well as the comparison with the related organism S. coelicolor allowed the identification of the phosphate regulon and the genes responsible for the replacement of cell wall teichoic acids with phosphate-free teichuronic acids.

## MATERIALS AND METHODS

### Strain cultivation and measurements.

Frozen cell stocks of *P. rosea* ATCC 53733 were routinely precultivated in 10 ml D-Seed (20 g/liter soluble starch, 5 g/liter peptone, 3 g/liter yeast extract, 2 g/liter meat extract, 2 g/liter soybean meal, 1 g/liter CaCO_3_ [pH 7.0]) at 30°C for 2 days at 200 rpm in 50-ml baffled flasks. Ten milliliters of preculture was transferred in 100 ml of medium C (35 g/liter soluble starch, 10 g/liter dextrose, 5 g/liter hydrolyzed casein, 3.5 g/liter meat extract, 8 g/liter yeast extract, 3.5 g/liter soybean meal, 2 g/liter CaCO_3_, 3.5 μg/ml CoCl_2_, 0.05% polyethylene glycol [PEG] [pH 7.2]) using 500-ml baffled flasks. In the case of phosphate and iron supplementation experiments, medium C was added with either 1 or 5 mM phosphate or with 0.25 or 1.2 mM FeCl_3_. Flasks were incubated at 30°C at 200 rpm. At the selected time points (15, 24, 39, 48, and 63 h), 1 ml was withdrawn from each flask and analyzed for cell weight, pH, and glucose concentration, while 0.5 ml was used for GE2270A production as described below. In supplementation experiments, phosphate amount was also measured. Wet cell weight at different time points was used to determine biomass accumulation. Accordingly, 1 ml was collected in a preweighed Eppendorf tube and centrifuged at 13,200 rpm for 2 min. The supernatant was discarded, and the remaining pellet was weighed. Glucose concentration was measured using a GM8 Micro-State (Analox Instruments) according to the manufacturer’s instructions, using 20 g/liter glucose as the standard. At the same time, samples for the transcriptome and metabolome extraction were collected (see below). GE2270A levels were measured by mixing 500-μl culture with an equal volume of acetonitrile in an Eppendorf tube and keeping the tube in a thermomixer at 1,400 rpm for 10 min at 40°C. Then the tube was centrifuged at 13,200 rpm for 2 min, and 20 μl of the resulting supernatant was analyzed by liquid chromatography-mass spectrometry (LC-MS) on a Dionex UltiMate 3000 coupled with an LCQ Fleet mass spectrometer equipped with an electrospray interface (ESI) and a tridimensional ion trap. The column was an Atlantis T3 C_18_ column (5 μm × 4.6 mm × 50 mm) maintained at 40°C at a flow rate of 0.8 ml/min. The aqueous phase (phase A) was 0.1% HCOOH, and the organic phase (phase B) was acetonitrile. The gradient was a 11-min multistep program that consisted of 10, 10, 95, 95, 10, and 10% phase B at 0, 1, 7, 9, 10, and 11 min, respectively. The UV detector was a diode array acquiring between 190 and 600 nm. GE2270A quantification was done at 310 nm. The *m/z* range was 110 to 2,000, and the ESI conditions were as follows: spray voltage of 3,500 V, capillary temperature of 275°C, sheath gas flow rate at 35 units, and auxiliary gas flow rate at 15 units. Phosphate concentration was measured using Merck Spectroquant phosphate test (*o*-phosphate), following the manufacturer’s instructions.

### Sample collection and extraction for metabolomics.

Three biological replicates of exometabolome and endometabolome samples were collected at five different time points (see above). In addition, an extra sample of medium before inoculation was collected as a control for the exometabolome analysis. For the whole-broth extraction, 1 ml of the culture was extracted by adding the same volume of acetonitrile (ACN) (ACN-H_2_O, 1:1) and mixed by vortexing. Aliquots of 200 μl were centrifuged for 10 min (4°C, at 4,500 rpm) and dried in Speedvac. The dried cell extracts were stored at −80°C until LC-MS analysis. For exometabolome 1 ml of culture medium was collected, centrifuged at 5,000 × *g* for 10 min and then subjected to a flash freezing in liquid nitrogen for 1 min. After thawing (on ice), aliquots of 200 μl of the sample were dried in Speedvac at room temperature. The dried samples were stored at −80°C until LC-MS analysis. For the endometabolome, 10 ml of a cold (−48°C) quenching solution (60% methanol) was added to 5 ml of bacterial culture, and the solution was centrifuged at 5,000 × *g* for 10 min (−4°C). Next, the supernatant was discarded, and 1 ml of a cold (−48°C) extraction solution (80% methanol) was added to the cell pellet which was then transferred to an Eppendorf tube. Metabolites were extracted by three freeze-thaw cycles in liquid N_2_ (i.e., flash frozen in liquid N_2_ for 1 min, thawed on ice, and vortexed). Then the samples were centrifuged at maximum speed for 5 min (−9°C), and 200-μl aliquots of supernatant dried in Speedvac at room temperature. The dried cell extracts were stored at −80°C until LC-MS analysis. On the day of the analysis, the samples were thawed and reconstituted in 200 μl of 20% methanol solution. The samples were vortexed and sonicated for 15 min and analyzed.

### Analyses of the acetonitrile extracts from the whole broth by LC-MS.

LC-UV-MS analysis was performed on an Agilent 1100 single quadrupole LC-MS system, using an Atlantis T3 column (5 μm, 4.6 × 100 mm), maintained at 40°C and with a flow rate of 1 ml/min. Solvent A consisted of 0.1% HCOOH in water, and solvent B was 0.1% HCOOH in acetonitrile. The elution of solvent B started at 5% for 2 min, and then it was increased to 100% within 11 min. This composition was maintained for 3 min, after which the elution of solvent B was decreased to 5% within 1 min. To reequilibrate the system, the elution of solvent B was held at 5% for 3 min. Full diode array UV scans from 100 to 900 nm were collected in 4-nm steps at 0.25 s/scan. Ionization of the eluting solvent was obtained using the standard Agilent 1100 ESI source adjusted to a drying gas flow of 11 liters/min at 325°C and a nebulizer pressure of 40 lb/in^2^ gauge (psig). The capillary voltage was set at 3,500 V. Mass spectra were collected as full scans from 110 *m/z* to 1,500 *m/z*, with one scan every 0.77 s, in both positive and negative modes. LC-LRMS (low-resolution mass spectrometry) was employed for quantification of the targeted compound GE2270A at 310 nm. High-resolution electrospray ionization mass spectrometry (HRESIMS) spectra were acquired using a Bruker maXis QTOF mass spectrometer coupled to the same high-pressure liquid chromatography (HPLC) system as described above. The mass spectrometer was operated in positive ESI mode. The instrumental parameters were 4 kV capillary voltage, drying gas flow of 11 liters/min at 200°C, and nebulizer pressure of 2.8 bar.

### LC-MS data acquisition.

The cell extracts were analyzed by Q Exactive Plus coupled to an Ultimate 3000 ultrahigh-performance liquid chromatography (UHPLC) (ThermoFisher, UK) equipped with a Hypersil Gold C_18_ reversed-phase HPLC column (3 μm, 2.1 mm, 100 mm; catalog no. 25003-102130; ThermoFisher, UK). The mobile phase consisted of solvent A (water plus 0.1% formic acid) and solvent B (methanol plus 0.1% formic acid). The flow gradient was programmed to equilibrate at 95% solvent A for 2 min, followed by a linear gradient to 95% solvent B over 8 min, held at 95% solvent B for 2 min, then followed by a return to 95% solvent A in 0.25 min and held at 95% solvent A for a further 2 min. The column was maintained at 40°C, and samples were chilled in the autosampler at 4°C. The flow rate was set at 0.4 ml/min. The sample injection volume was 5 μl. Blank injections were analyzed at the start and end of the analytical batch to assess the carryover. In addition, pooled quality control (QC) samples were analyzed at every sixth injection to assess for analytical drift over time. The sample sequence was randomized. Data were acquired in full MS mode in the scan range of 90 to 1,350 *m/z*, with a resolution of 70,000, an AGC target of 3e6, and a maximum integration time of 200 ms. The samples were analyzed in positive and negative mode in separate acquisitions.

### Metabolomics data analysis.

Raw data files from the Q Exactive were converted into the mzML format by the ProteoWizard MS converter. Data analysis was performed with the use of mzMatch, a modular, open-source, and platform-independent data processing pipeline for metabolomics LC-MS data written in the Java language implemented in R (53). Noise removal, signal filtering, and peak matching steps were performed. The detected features were grouped according to their likelihood to be associated with one single molecule, and only the most intense peak is considered for the subsequent statistical analysis. Putative annotation for the detected features was performed with the Integrated Probabilistic Annotation (IPA) (44), using an *ad hoc* database, including the KEGG database and the known GE2270A congeners (5). GE2270A was identified against the molecular weight and retention time of a standard.

### RNA purification, quality control, and sequencing.

Culture samples were collected at 15, 24, 39, 48, and 63 h from three *P. rosea* independent cultures grown in medium C and stabilized with 2 volumes of RNAProtect Bacteria reagent (Qiagen, DE) according to the manufacturer’s instructions. For RNA extraction, cell pellets were suspended in 0.17 ml of lysozyme (15 mg/ml) and incubated at 30°C for 10 min. Then, each suspension was transferred to a tube of lysing matrix B beads (MP Biomedicals, UK) containing 0.6 ml of RLT buffer (Qiagen, DE) supplemented with β-mercaptoethanol (100:1). Total cell lysis was achieved by two pulses at 6.5 m/s, 30 s in a FastPrep instrument (MP Biomedicals, UK); samples were placed on ice between pulses. RNA was extracted with a mixture of acid phenol, chloroform, and isoamyl alcohol (25:24:1). Total RNA was purified according to the manufacturer’s instructions with Direct-zol RNA MiniPrep Plus columns (Zymo Research, USA). The purity and concentration of RNA preparations were estimated using a NanoDrop 1000 (Thermo Scientific, USA). The integrity of RNA molecules was assessed through capillary electrophoresis with RNA Nano chips and a Bioanalyzer 2100 system (Agilent Technologies, USA). RNA preparations were of high purity, concentration (>1 μg/μl) and integrity (RNA integrity number [RIN] of >9.0). rRNA depletion, TruSeq library preparation, and RNA sequencing were conducted by vertis Biotechnologie AG (Freising, DE). Briefly, rRNA molecules were depleted using the Ribo-Zero rRNA removal kit for bacteria (Illumina). Then, RNA samples were fragmented using ultrasound (4 pulses of 30 s each at 4°C). After adapter ligation to the 3′ ends, first-strand cDNA synthesis was performed using Moloney murine leukemia virus (M-MLV) reverse transcriptase and the 3′ adapter as a primer. After cDNA purification, the 5′ adapter was ligated to the 3′ end of the antisense cDNA. The resulting cDNA was amplified to about 10 to 20 ng/μl using a high-fidelity DNA polymerase and 13 PCR cycles. The cDNA was purified using the Agencourt AMPure XP kit (Beckman Coulter Genomics). Samples were pooled in approximately equimolar amounts and fractionated in a preparative agarose gel to recover molecules in the range of 180 to 550 bp. Single-end sequencing was conducted on an Illumina NextSeq 500 system of 75-bp read length.

### RNAseq bioinformatics analysis.

Fastq files containing the raw reads were processed with BBDuk and BBMap programs (B. Bushnell, sourceforge.net/projects/bbmap/). BBDuk served to remove adapter sequences (parameters: ′ktrim=r k=23 mink=11 hdist=1′) and, in a second run, to filter reads by length and quality (′minlen=20 maq=10′). BBMap, run in local mode (′slow=t ambiguous=random maxindel=1 strictmaxindel=t local=t minid=0.8′), served to map the filtered reads to the *P. rosea* genome (GenBank accession no. NZ_JPMW00000000 [18]). Reads mapped on rRNA genes (coordinates, NZ_JPMW01000001.1, 1778004 to 1783349, both strands) were remove with program split_bam.py of RSeQC package (54). Final library sizes were in the range 7.6 × 10^6^ to 10.6 × 10^6^ reads. Alignment and genome data were processed in the R environment (version 3.6) using Bioconductor packages Rsamtools (2.2.3), GenomicFeatures (1.38.2), and GenomicAlignments (1.22.1) (55). The summarizeOverlaps function of GenomicAlignments with mode “Union” was used to count reads mapped to annotated genes (7,457 protein-coding genes, 269 pseudogenes, 63 tRNA, 3 rRNA, 1 transfer messenger RNA [tmRNA], 2 noncoding RNA [ncRNA]). The transcript per million (TPM) values (56) were calculated from read counts using in-house spreadsheets. Bioconductor package DESeq2 (version 1.26.0) (57) was used to normalize the read counts with respect to library size and to transform the normalized count data in the log_2_ scale with the regularized logarithm method, rlog function. The rlog values were used for the differential expression analysis with an ANOVA test, and the obtained *P* values were corrected for multiple testing with the Benjamini-Hochberg method (43).

### Construction of the genome-scale metabolic model.

The genome-scale metabolic model here introduced was built using the COBRApy toolbox (58). Here, we constructed the first draft genome-scale metabolic model using a comparative approach. The recently published genome-scale model for Streptomyces coelicolor (iAA1259 [46]) was used as starting point for the model. In fact, S. coelicolor is the closest phylogenetically related organism to *P. rosea* for which a well-curated and validated genome-scale model exists. By using this model as a template, all the genes that did not show any homologue or orthologue in *P. rosea* were removed from the model, together with the related reactions and metabolites. The exact biosynthetic pathway for the biosynthesis of GE2270A is not yet known. Consequently, this pathway is modeled as a single global reaction. Similarly, to B. subtilis and S. coelicolor, the transcriptomics data suggest that during phosphate starvation *P. rosea* replaces the cell wall teichoic acids with teichuronic acids. Hence, an alternative biomass reaction has been included in the model, together with the reactions needed for the biosynthesis of teichuronic acid. The teichuronic acid considered is the one first isolated from the walls of Bacillus licheniformis 6346 and characterized as a polymer containing *N*-acetylgalactosamine and d-glucuronic acid in equal proportions (47). The metabolite defined in the model consists of 25 repeating units of such polymer. In order to allow its production, the model required the addition of the reaction for the biosynthesis of teichuronic acid from UDP-*N*-acetylglucosamine and UDP-d-glucuronate. The production of UDP-*N*-acetylglucosamine was obtained by the addition of the UDP-*N*-acetylglucosamine 4-epimerase reaction. In B. subtilis, this reaction is catalyzed by *galE* (48). An orthologue of this gene is present in the *P. rosea* genome (locus tag EV45_RS04840).

### Definition of the parameter distribution.

The ensemble modeling approach described in the “Genome-scale metabolic model” section considers seven parameters: maximum O_2_ uptake rate, *V*_max_ and *K_m_* for glucose uptake, *V*_max_ and *K_m_* for phosphate uptake, and *V*_max_ and *K_m_* for teichoic acid consumption. To the best of our knowledge, no measurement of the maximum O_2_ uptake rate was reported for *P. rosea*. Varma and Palsson (59) reported the maximum oxygen utilization rate for E. coli W3110 to be 15 mmol/gDW/h. Given this reference point, we defined the distribution for this parameter as a log-normal distribution where the mode is equal to 12 mmol/gDW/h and 95% of the values lie between 6.7 and 15 mmol/gDW/h. In the same paper, Varma and Palsson (59) also measured the maximum glucose utilization rate under aerobic conditions (10.5 mmol/gDW/h) and under anaerobic conditions (18.5 mmol/gDW/h) for E. coli W3110. Since we are assuming that in our *in silico* experiment *P. rosea* grows under aerobic conditions, we defined the distribution for the glucose *V*_max_ parameter as a log-normal distribution where the mode is equal to 10 mmol/gDW/h and 95% of the values lie between 9.6 and 15 mmol/gDW/h. Boles and Hollenberg (60) measured the glucose *K_m_* values for several sugar transporters found in Saccharomyces cerevisiae, Kluyveromyces lactis, Schizosaccharomyces pombe, and Pichia stipitis. All the transporters with high affinity with glucose have a *K_m_* value between 1.5 and 6 mmol/liter. Therefore, we defined the distribution for the glucose *K_m_* parameter as a log-normal distribution where the mode is equal to 3 mmol/liter and 95% of the values lie between 1.5 and 6 mmol/liter. Nieselt et al. (61) monitored the growth of S. coelicolor in a medium designed to be phosphate limited. They also monitored the phosphate extracellular concentration, which allowed to have a reasonable estimate for the *V*_max_ and *K_m_* parameters associated with phosphate. Hence, we defined the distribution for the phosphate *V*_max_ parameter as a log-normal distribution where the mode is equal to 0.13 mmol/gDW/h and 95% of the values lie between 0.156 and 0.108 mmol/gDW/h. We defined the distribution for the phosphate *K_m_* parameter as a log-normal distribution where the mode is equal to 0.065 mmol/liter and 95% of the values lie between 0.054 and 0.078 mmol/liter. Regarding the usage of teichoic acid as a reserve of phosphate, very little is reported in the literature. Grant (32) reported that during phosphate starvation, B. subtilis lost 66% of the phosphate in the cell wall within 5 h. Therefore, we estimated the *V*_max_ and *K_m_* parameters associated with teichoic acid to be consistent with this observation. We defined the distribution for the teichoic acid *V*_max_ parameters as a log-normal distribution where the mode is equal to 10 mmol/gDW/h and 95% of the values lie between 1 and 100 mmol/gDW/h. We defined the distribution for the phosphate *K_m_* parameter as a log-normal distribution where the mode is equal to 0.015 mmol/liter and 95% of the values lie between 0.0015 and 0.15 mmol/liter. The obtained parameter distributions are reported in Fig. S10 at https://github.com/francescodc87/Multi-omics-study-of-Planobispora-rosea.

### Data availability.

All supplemental data can be found at https://github.com/francescodc87/Multi-omics-study-of-Planobispora-rosea. Metabolomics processed data can be found in Supplementary files 7, 8, 9, and 10. The original data from the project are accessible for analysis on MORF (https://morf-db.org/projects/TOPCAPI/P-rosea), an entirely browser-based multi-omics tools (20). This enables full analysis of the transcriptomics data and tools for analysis of the published genome. Also, the genome-scale metabolic model, which can also be found in Supplementary file 11, is hosted in the MORFlux FBA Tool for direct access, download, and analysis.
